# Three-dimensional cephalometric outcome predictability of virtual orthodontic-surgical planning in surgery-first approach

**DOI:** 10.1186/s40510-022-00448-x

**Published:** 2022-12-30

**Authors:** Giovanni Badiali, Mirko Bevini, Chiara Gulotta, Ottavia Lunari, Serena Incerti Parenti, Marco Pironi, Alberto Bianchi, Pietro Felice, Claudio Marchetti

**Affiliations:** 1 IRCCS Azienda Ospedaliero-Universitaria di Bologna, Via Albertoni 15, Bologna, Italy; 2 Department of Biomedical and Neuromotor Sciences, University of Bologna, Bologna, Italy; 3 University of Bologna, Bologna, Italy; 4 Maxillofacial Surgery Unit, Department of Surgery and Surgical Specialties, Azienda Ospedaliero Universitaria “Rodolico - S. Marco”, University of Catania, Catania, Italy; 5 Private Practice, Rimini, Italy

## Abstract

**Objectives:**

The aim of this study is to introduce a novel 3D cephalometric analysis (3DCA) and to validate its use in evaluating the reproducibility of virtual orthodontic-surgical planning (VOSP) in surgery-first approach (SF) comparing VOSP and post-operative outcome (PostOp).

**Methods:**

The cohort of nineteen patients underwent bimaxillary orthognathic surgery following the VOSP designed in SimPlant O&O software by processing cone-beam computed tomography (CBCT) scans and intraoral digital scanning of the dental arches. Said records were re-acquired once the post-operative orthodontic treatment was completed. The 3DCA was performed by three expert operators on VOSP and PostOp 3D models. Descriptive statistics of 3DCA measures were evaluated, and outcomes were compared via Wilcoxon test.

**Results:**

In the comparison between cephalometric outcomes against planned ones, the following values showed significant differences: Wits Index, which suggests a tendency towards skeletal class III in PostOp (*p* = 0.033); decreased PFH/AFH ratio (*p* = 0.010); decreased upper incisors inclination (*p* < 0.001); and increased OVJ (*p* = 0.001). However not significant (*p* = 0.053), a tendency towards maxillary retroposition was found in PostOp (A/McNamara VOSP: 5.05 ± 2.64 mm; PostOp: 4.1 ± 2.6 mm). On average, however, when McNamara’s plane was considered as reference, a tendency to biprotrusion was found. Upper incisal protrusion was greater in PostOp as an orthodontic compensation for residual maxillary retrusion (VOSP: 5.68 ± 2.56 mm; PostOp: 6.53 ± 2.63 mm; *p* = 0.084). Finally, the frontal symmetry in relation to the median sagittal plane decreased in craniocaudal direction.

**Limitations:**

A potential limit of studies making use of closest point distance analysis is represented by the complexity that surgeons and orthodontists face in applying this three-dimensional evaluation of SF accuracy/predictability to everyday clinical practice and diagnosis. Also, heterogeneity and limited sample size may impact the results of the study comparison.

**Conclusions:**

The presented 3DCA offers a valid aid in performing VOSP and analysing orthognathic surgery outcomes, especially in SF. Thanks to the cephalometric analysis, we found that surgery-first approach outcome unpredictability is mainly tied to the sagittal positioning of the maxilla and that the transverse symmetry is progressively less predictable in a craniocaudal direction.

## Introduction

In orthognathic surgery, surgery-first approach (SF) is an increasingly popular alternative to the conventional orthodontics-first approach (OF), with a growing body of the literature and interest from orthodontists, surgeons, and patients themselves. Alfaro et al. reported 18.8% of their orthognathic cases are treated by means of SF [1].

When compared to standard OF, the SF approach is tied to higher uncertainty in terms of both morphological and occlusal results; the main drawback of SF is the challenge in predicting combined skeletal and dental movements, since teeth cannot guide the planning of skeletal movements [2]. Therefore, SF requires a clear definition of the dentoskeletal deformity, a personalized orthodontic-surgical planning and accurate reproduction of the surgical planning in theatre and orthodontics post-operatively. Only if these conditions are met, the outcome can be both accurate and predictable [3].

To overcome this limitation, recent improvements in both orthodontics and surgery can be integrated to increase the accuracy of said approach [4]. Alfaro et al. [5] described a specific orthodontic and surgical protocol for SF, discussing the benefits and limitations of this treatment. We also presented a new computer-assisted method, which combines virtual orthodontic planning (VOP) and virtual surgical planning (VSP) into a virtual orthodontic-surgical planning (VOSP) to simulate the treatment [6].

By overlapping three-dimensional surfaces in a post-operative analysis, the VOSP demonstrated to be reliably transferred to the patient. However, a limit of studies making use of surface comparison evaluation is the complex, and at times not univocal, interpretation of results given by such method [7].

With its many facets and uncertainties, both on the skeletal and dental side, SF outcome analysis is a perfect test bench for a diagnostic tool such as three-dimensional cephalometry (3DCA), which carries the advantages of allowing a more standardized interpretation and a stronger correlation to clinical outcomes.

On a literature basis, we selected 3D cephalometric measurements which transpose 2D cephalometry in a three-dimensional environment [8, 9], adding new measurements which are purely three-dimensional [10–13]. Thus, we here introduce a 3DCA based on our clinical practice, by means of which we analysed a cohort of SF patients, with the aim of deepening our comprehension of the uncertain aspects in this approach.

## Materials and methods

### Study design

Nineteen consecutive Caucasian patients who presented with dentofacial deformities at the Oral and Maxillofacial Surgery Unit of the S. Orsola University Hospital in Bologna (Italy) were enrolled between 2013 and 2019 and treated with a SF approach, according to the inclusion/exclusion criteria listed in Table 1.

**Table 1:** Inclusion and exclusion criteria

Inclusion criteria	Exclusion criteria
18 + years old Caucasian ethnicity Operated by the same surgical team Available pre- and post-surgical CBCT Mono- or bimaxillary surgery	Any ethnicity other than Caucasian Syndromic patients Ancillary soft or hard tissue surgery performed

This monocentric prospective protocol was approved in 2013 by our local ethics committee. The study conformed to the principles of the Declaration of Helsinki. Written informed consent was obtained from all patients upon enrolment in the trial.

Eleven males and eight females were enrolled in the study, with mean age 26.5 years (range 18–55 years). The cohort was composed of 65% class 3, 30% class 2, and 5% class 1 patients.

Each patient underwent the workflow described below:

### Data acquisition

Patients underwent CBCT scan (VGi; NewTom, Verona, Italy) with a 19 × 19 field-of-view (FOV) and intraoral digital scanning of the dental arches (Trios; 3Shape, Copenhagen, Denmark) in the pre-operative phase.

The DICOM datasets and the STL files were processed using the SimPlant O&O platform (Dentsply-Sirona; York, PA, USA), to produce an accurate 3D model of the patient’s hard and soft tissues. The 3DCA completed the diagnostic process; VOSP was subsequently performed on the 3D models.

### 3D cephalometry

3D cephalometry was performed by three expert operators (two orthodontists and one maxillofacial surgeon) for each patient using the Simplant O&O Software.

The three analyses performed were compared via a Friedman test for inter-operator concordance. Once the concordance was ascertained, the average of each value was used in further comparisons.

The authors developed a specific 3DCAderived from traditional 2D analyses (Ricketts, McNamara, Arnett, Tweed) plus several measurements regarding both vertical and transverse symmetry, which are only possible in three-dimensional studies, with the aim of identifying an array of clinically relevant landmarks and measurements in orthodontic-surgical patient treatment [14].

**Fig. 1:**
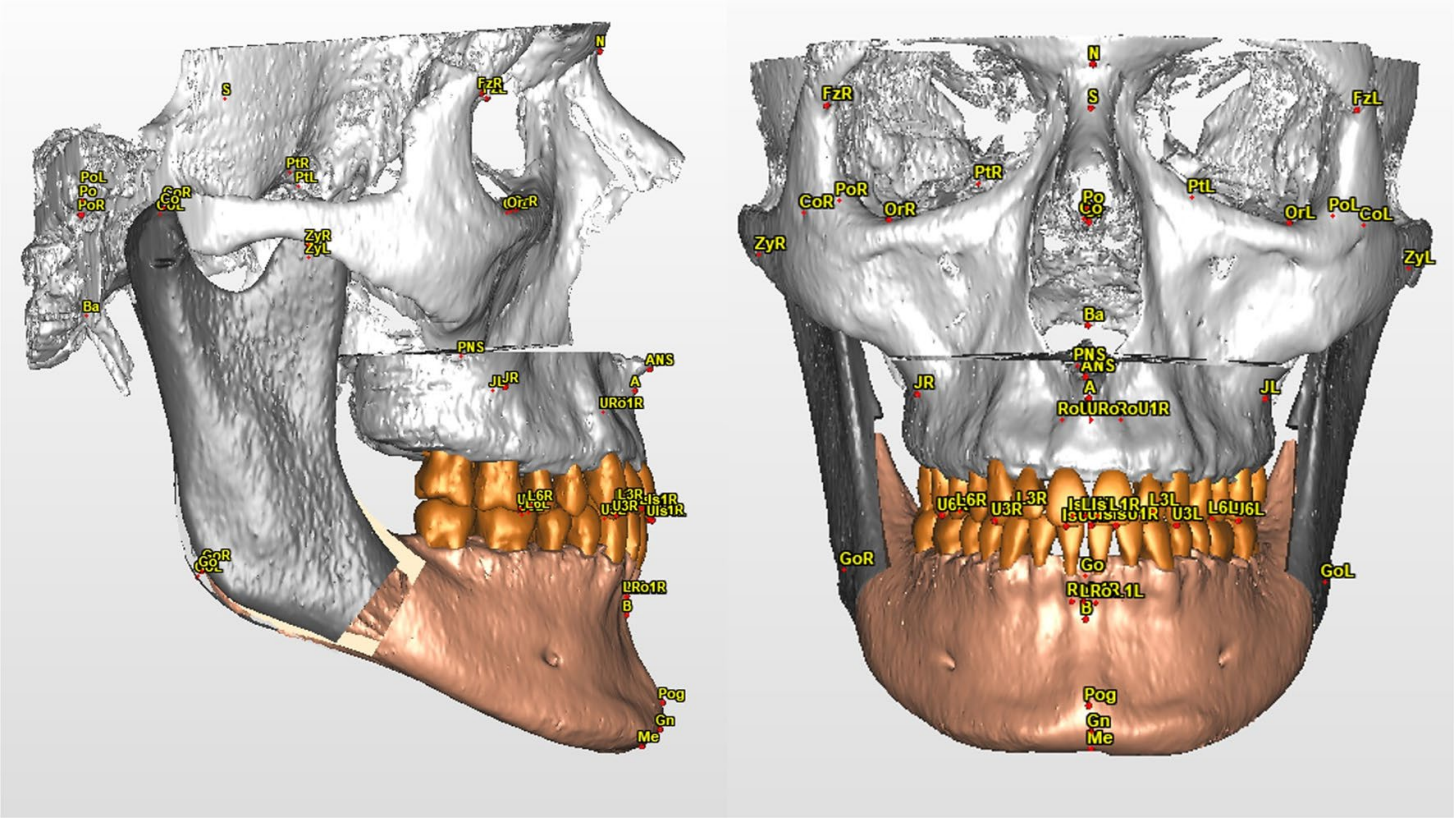
Skeletal and dental cephalometric landmarks in a VOSP representation

The analysis consists of 42 skeletal and dental landmarks, listed in Table 2, that the operator selects on patient’s 3D reconstruction (Fig. 1) and on the three-plane reslicing of the CT images.

**Table 2:** Skeletal and dental cephalometric landmarks

Point	Description
Nasion (N)	Midpoint of the frontonasal suture
Sella (S)	Central point of the hypophyseal fossa (sella turcica)
Porion (Po-L/R)	Higher point of the external ear canal, right and left
Orbitale (Or-L/R)	Anteroinferior point of each orbital rim
Anterior Nasal Spine (ANS)	Most anterior median point of the anterior nasal spine
Posterior Nasal Spine (PNS)	Most posterior median point of the posterior nasal spine
Point A	Maximum concavity point on the median line of the maxillary alveolar process
Point B	Maximum concavity point on the median line of the mandible
Pogonion (Pog)	Most anterior point on the medial line of the mandibular symphysis
Menton (Me)	Lower point of the chin on the median line
Gnathion (Gn)	Lower anterior point on the median line of the mandibular symphysis
Basion (Ba)	Most anterior point of the foramen magnum
Gonion (Go-L/R)	Most inferior, posterior, and lateral point on the angle of the mandible
Frontozygomatic (Fz-L/R)	Most medial and anterior point of each frontozygomatic suture
Zygion (Zy-L/R)	Lateral point of each zygomatic arch
Jugale (J-L/R)	Lateral point of the maxillary upright
Condylion (Co-L/R)	Most posterior-superior point of each condyle on the sagittal plane
Pterion (Pt-L/R)	Point of intersection between the round foramen and the pterygomaxillary fossa, left and right
Upper first Left/Right (U1-L/R)	Most occlusal point of the upper central incisors, left and right
Upper first Left/Right Root (U1Ro-L/R)	Root apex of upper central incisors, right and left
Upper third Left/Right (U3-L/R)	Cusp of the upper right and left canines
Upper sixth Left/Right (U6-L/R)	Mesiobuccal cusp of the superior first molars, right and left
Lower first Left/Right (L1-L/R)	Most occlusal point of the lower central incisors, right and left
Lower first Left/Right Root (L1Ro-L/R)	Root apex of lower central incisors, right and left
Lower third Left/Right (L3-L/R)	Cusp of lower right and left canines
Lower sixth Left/Right (L6-L/R)	Mesiovestibular cusp of the first lower molars, right and left

**Fig. 2:**
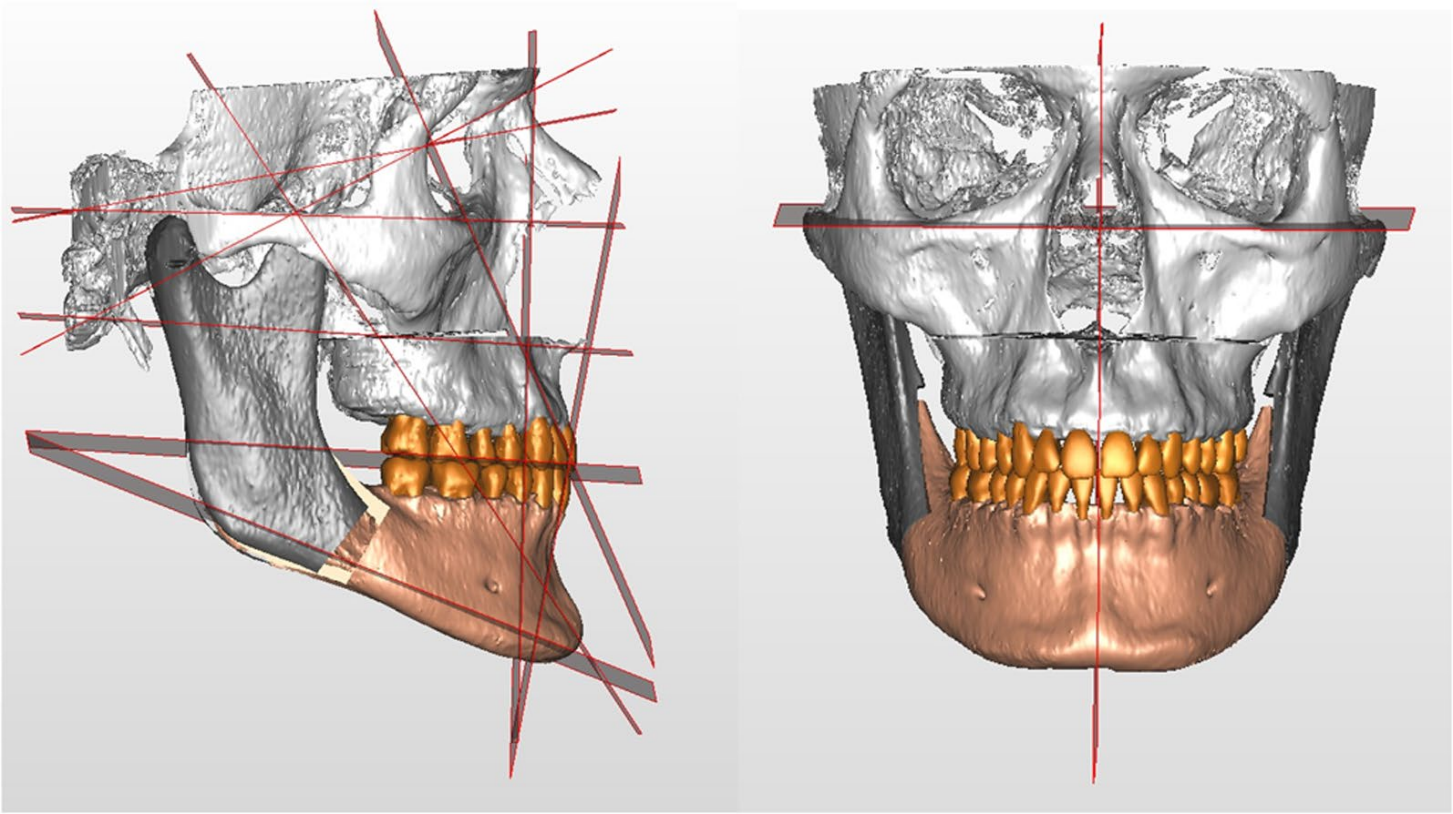
Example of cephalometric reference planes in a VOSP representation

Part of these points are used by the software to generate reference planes, which are listed in Table 3 and shown in Fig. 2. Based on these points and planes, linear and angular measurements are computed by the software according to the rules set by the clinician.

**Table 3:** Cephalometric reference planes

Plane	Description
Sagittal plane	Plane passing through Nasion and Basion and perpendicular to Frankfurt plane
Plane of Frankfurt (FH Plane):	Plane passing through Porion and Orbitale and perpendicular to the Sagittal plane
Vertical Plane (McNamara plane):	Plane perpendicular to Frankfurt plane passing through Nasion
Occlusal plane (Occlusal Plane)	Plane passing through the mesiovestibular cusps of the first upper right and left molars and the inter-incisive point
Mandibular Plane (Mandibular Plane)	Plane passing through Go-R, Go-L and Me
Facial axis plane (Facial Axis Plane)	Plane passing through Pt-R, Pt-L and Gn
Upper incisors axis plane (Upper Incisors Axial Plane)	Plane passing through U1-L/R and U1Ro-L/R
Lower incisors axis plane (Lower Incisors Axial Plane)	Plane passing through L1-L/R and L1Ro-L/R
Plane A	Plane parallel to McNamara plane passing through point A
Plane B	Plane parallel to the McNamara plane passing through point B

#### Sagittal skeletal analysis

The sagittal skeletal analysis includes the so-called strictly sagittal parameters which identify the skeletal class, anterior/posterior position of the jaw, divergence, and facial type of the patient. It also includes sagittal symmetry values.

**Fig. 3:**
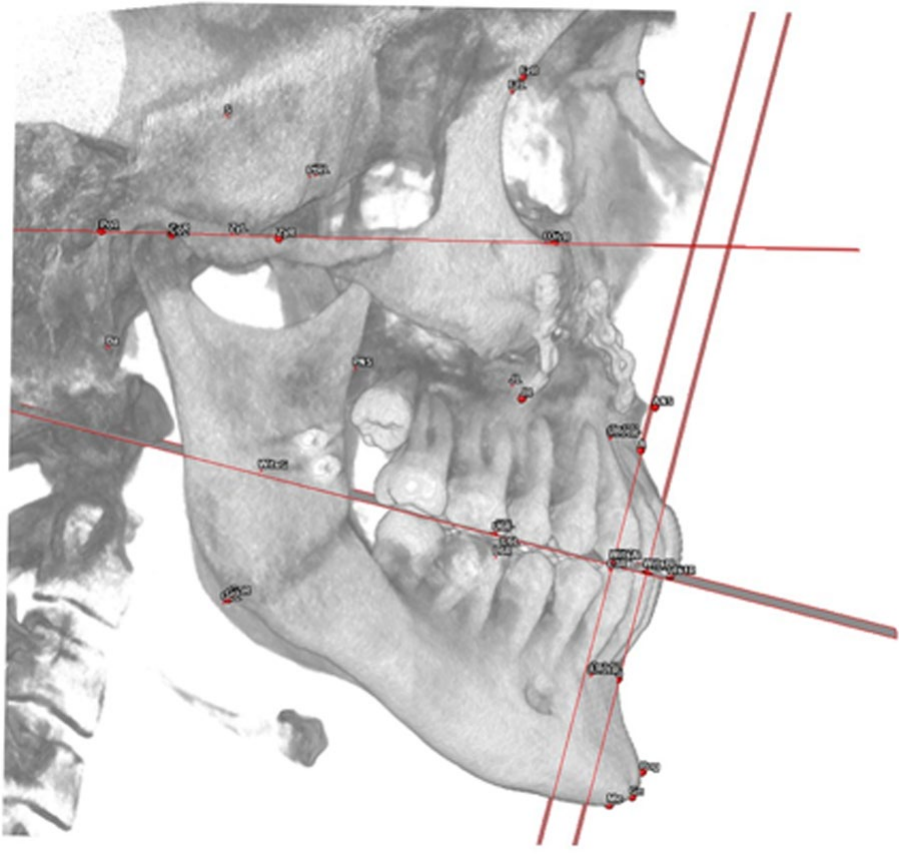
View of a sagittal skeletal analysis in a PostOp case

Moreover, the linear measurements of both sides (right and left) are compared computing the difference between the two (△) (Fig. 3, Table 4).

**Table 4:** Sagittal skeletal analysis: sagittal parameters and symmetry values

*Strictly sagittal parameters*	
WITS Index	Distance between point A and B projection on the occlusal plane (mm)
A, B, Pog/McNamara	Distance of points A, B and Pog from the McNamara plane (mm)
Facial Axis Angle	Angle between facial axis plane and the straight line combines points Ba and N (°)
Tweed Mandibular angle	Angle between the mandibular plane and the Frankfurt plane (°)
PFH/AFH	Ratio of posterior facial height to anterior facial height (Na-Me / Po-Go)
*Sagittal symmetry values*	
△ Maxillary Length	Difference in distance between point A and Co (left and right) (mm)
Mean Maxillary Length	Mean of point A—Co (left and right) distances (mm)
△ Mandibular Length	Difference in distance between Gn and Co (left and right) (mm)
Mean Mandibular Length	Mean of Gn-Co (left and right) distances (mm)
△ Md Body Length	Difference in distance between Md Body (left and right) (mm)
Mean Md Body Length	Mean of Md Body (left and right) distances (mm)
△ Gonial Angle	Difference between Gonial Angle (left and right) (°)
Mean Gonial Angle	Mean of Gonial Angle (left and right) angles (°)

#### Frontal skeletal analysis

**Fig. 4:**
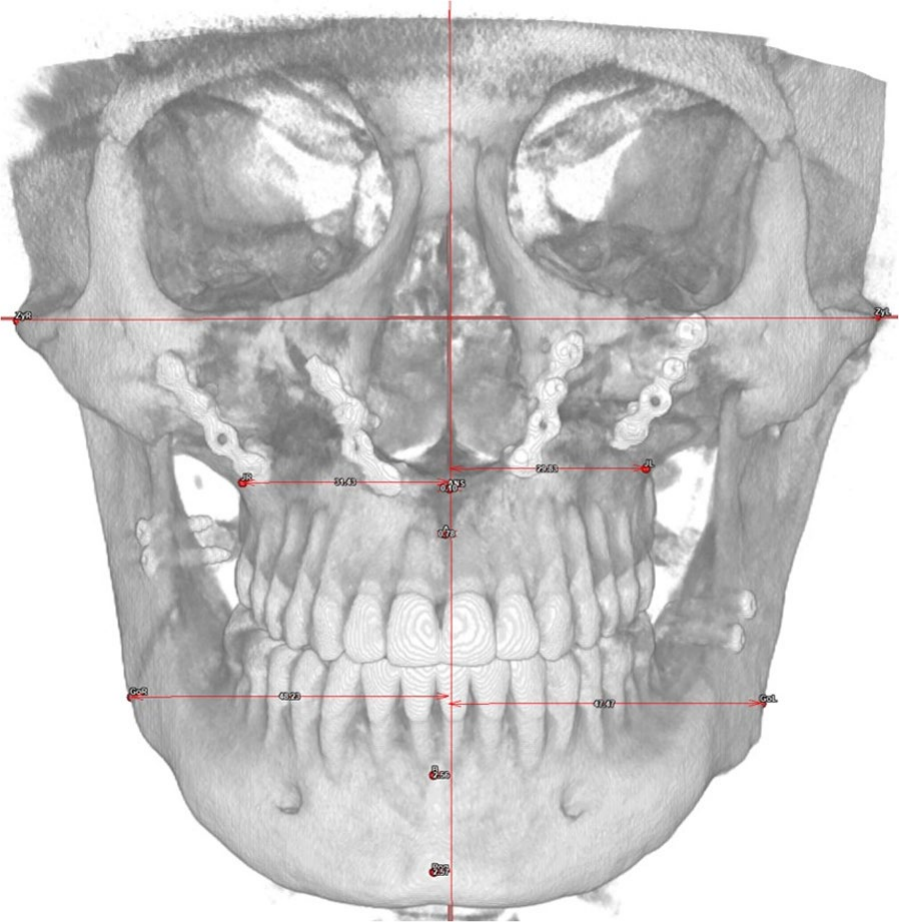
View of a frontal skeletal analysis in a PostOp case

The frontal skeletal analysis focuses on frontal symmetry, using both midline parameters, which represent the distances in millimetres of the points ANS, A, B and Pog from the sagittal plane and differences between distances of theoretically symmetrical points from the sagittal plane (Fig. 4, Table 5).

**Table 5:** Frontal skeletal analysis: frontal parameters and symmetry values

*Strictly frontal parameters*	
ANS–SagPL	Subnasal Deviation from the Sagittal Plane (mm)
A–SagPL	Maxillary Deviation from the Sagittal Plane (mm)
B–SagPL	Mandibular Deviation from the Sagittal Plane (mm)
Pog–SagPL	Mental Deviation from the Sagittal Plane (mm)
*Frontal symmetry values*	
△ Go-SagPL (L.R)	Difference in distance between Go (left and right) and Sagittal Plane (mm)
Mean Go-SagPL (L.R)	Mean of Go (left and right) distances from Sagittal Plane (mm)
△ J-SagPL (L.R)	Difference in distance between J (left and right) and Sagittal Plane (mm)
Mean J-SagPL (L.R)	Mean of J (left and right) andSagittal Plane distances (mm)
△ Zy-SagPL (L.R)	Difference in distance between Zy (left and right) and Sagittal Plane (mm)
Mean Zy-SagPL (L.R)	Mean of Zy (left and right) and Sagittal Plane distances (mm)

#### Vertical skeletal analysis

**Fig. 5:**
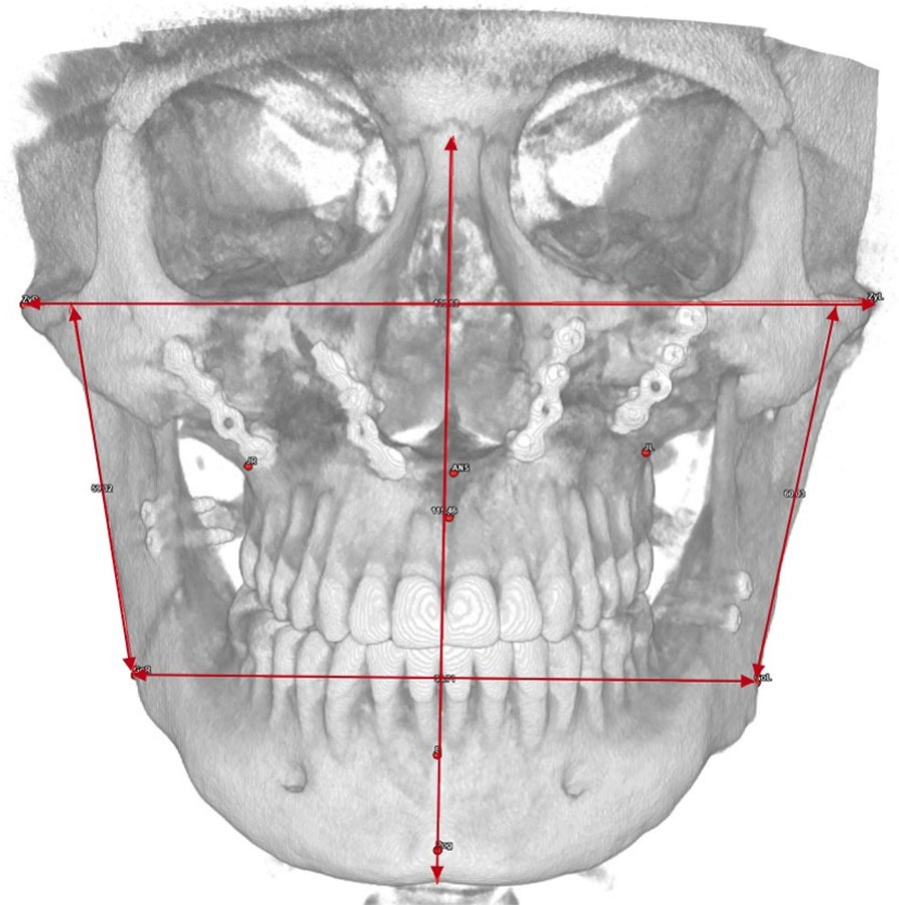
View of a vertical skeletal analysis in a PostOp case

The vertical skeletal analysis allows clinicians to evaluate the patient’s vertical proportions and classify them as a long / short face. It also includes vertical symmetry values. (Fig. 5, Table 6).

**Table 6:** Vertical skeletal analysis: vertical parameters and symmetry values

*Strictly vertical parameters*	
Facial ratio	Na-Me distance/Zy(L)-Zy(R) distance
Mandibular ratio	Co-Go distance/Go(R)-Go(L) distance
*Vertical symmetry values*	
△ Md Ramus length (L.R)	Difference in distance between Md Ramus length (left and right) (mm)
Mean Md Ramus length (L.R)	Mean of the two distances Md Ramus length (left and right) (mm)

#### Sagittal dento-alveolar analysis

**Fig. 6:**
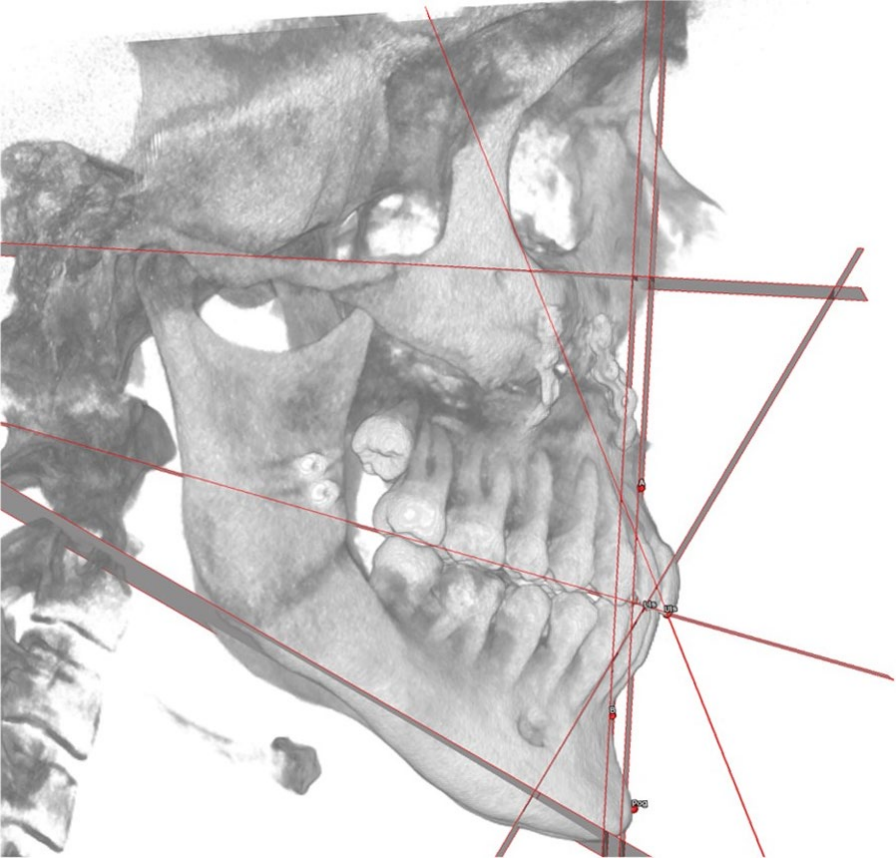
View of a sagittal dento-alveolar analysis in a PostOp case

The dento-alveolar sagittal analysis evaluates the incisors’ position in respect to the maxillary and mandibular bone, in respect to the occlusal plane and in respect to each other (Fig. 6, Table 7).

**Table 7:** Sagittal dento-alveolar analysis: sagittal parameters

Strictly sagittal parameters	
Incisal Protrusion	Distance between UIs and plane A (mm)
UIs/Occlusal plane angle	Angle between UIs axis and occlusal plane (°)
IMPA (Incisor-Mandibular plane angle)	Angle between LIs axis and Mandibular plane (°)
Overjet (OVJ)	Linear distance between incisal margin of the upper incisors and vestibular surface of the lower incisors (mm)
Overbite (OVB)	Linear distance between LIs incisal margin and projection of the incisal edge of the UIs (mm)
Occlusal plane inclination	Angle between occlusal plane and frankfurt plane (°)
LIs/B plane angle	Angle between LIs axis and plane B (°)

#### Frontal dento-alveolar analysis

**Fig. 7:**
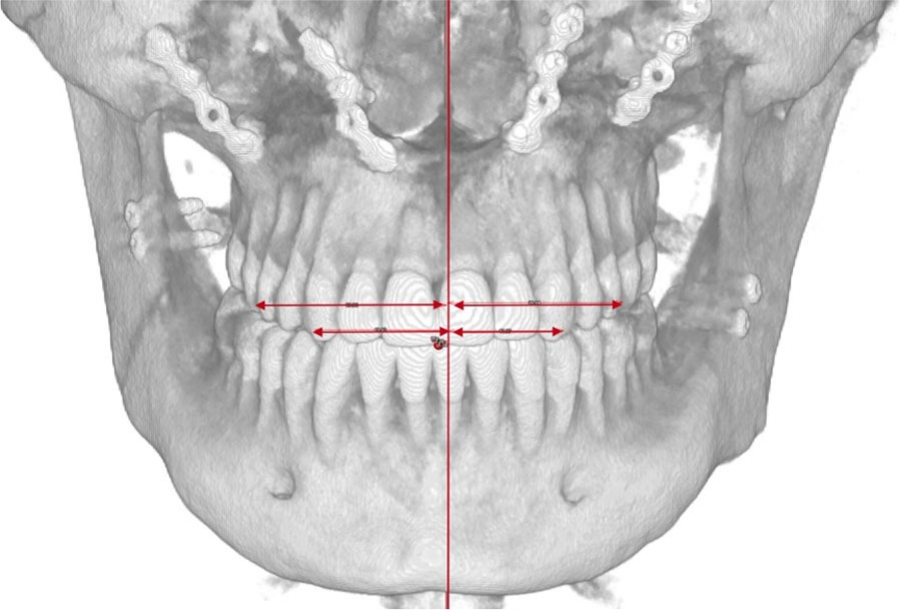
View of a frontal dento-alveolar analysis in a PostOp case

The dento-alveolar frontal analysis evaluates the distance between the inter-incisal line, canines and molars to the sagittal plane (Fig. 7, Table 8).

**Table 8:** Frontal dento-alveolar analysis: frontal parameters

Strictly Frontal parameters	
UIs-SagPl	Distance between UIs and sagittal plane (mm)
Lis-SagPl	Distance between LIs and sagittal plane (mm)
△ U3-SagPl	Difference in distance between U3 cusp (left and right) and sagittal plane (mm)
U3-SagPl Mean	Mean of the distances between U3 cusp (left and right) and sagittal plane (mm)
△ U6-SagPl	Difference in distance between U6 mesiovestibular cusp (left and right) and sagittal plane (mm)
U6-SagPl Mean	Mean of the distances of U6 mesiovestibular cusp (left and right) and sagittal plane (mm)

#### Vertical dento-alveolar analysis

**Fig. 8:**
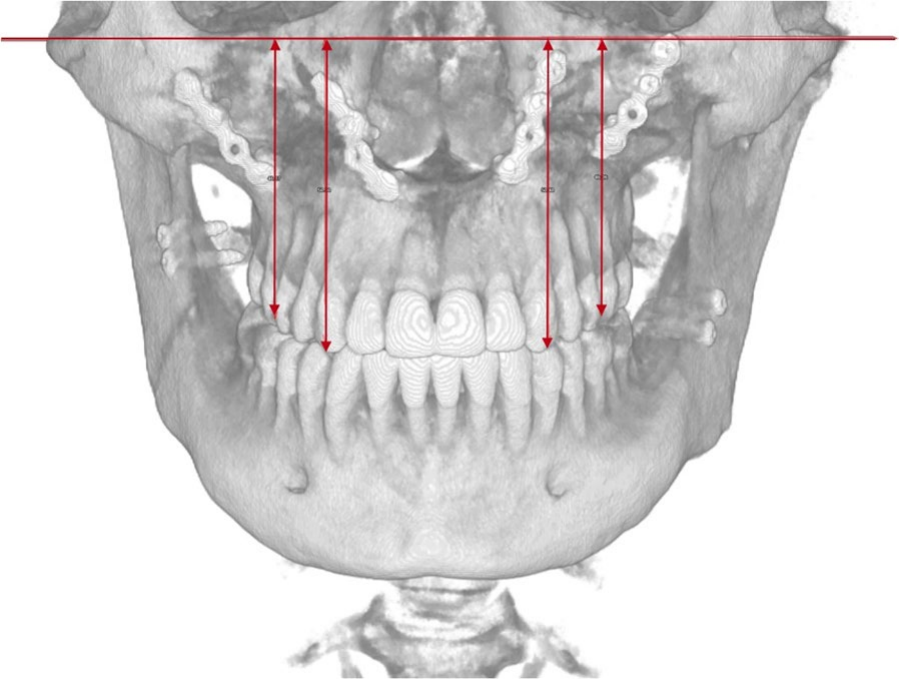
View of a vertical dento-alveolar analysis in a PostOp case

The dento-alveolar vertical analysis evaluates the vertical distance between the cusp of the upper canine and the mesiovestibular cusp of the first upper molar and the Frankfurt plane (Fig. 8, Table 9).

**Table 9:** Vertical dento-alveolar analysis: vertical parameters

Strictly vertical parameters	
U3-FH	Distance between U3 cusp (left and right) and FH (mm)
U6-FH	Distance between U6 mesiovestibular cusp (left and right) and FH (mm)
△ U3-FH	Difference in distance between U3 cusp (left and right) and FH (mm)
Mean U3-FH	Mean of the distances between U3 cusp (left and right) and FH (mm)
△ U6-FH	Difference between U6 mesiovestibular cusp (left and right) and FH (mm)
Mean U6-FH	Mean of the distances between U6 mesiovestibular cusp (left and right) and FH (mm)

### Virtual planning

#### Virtual orthodontic planning (VOP)

**Fig. 9:**
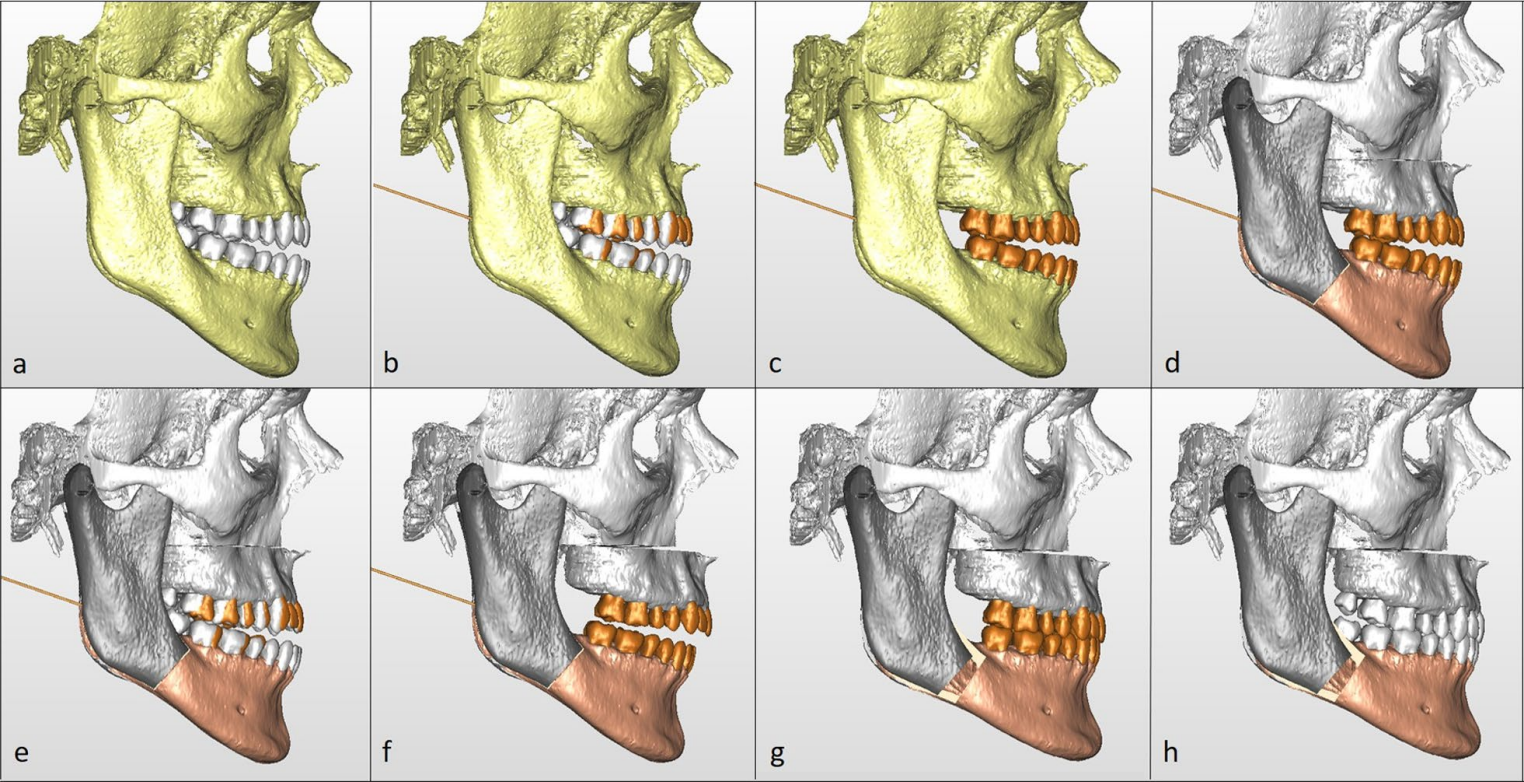
VOSP wokflow. Figures are labelled form top left to bottom right. **A**, Patient 3D reconstruction; **B**, The ideal virtual arches of both arches were registered on the skeletal base, superimposed on the native arches; **C**, Skeletal base with ideal virtual arches; **D**,**E**, Virtual osteotomy of the base of the facial skeleton with ideal virtual arch and both ideal and native; **F**, Virtual surgical plan: repositioning of the upper jaw, followed by the virtually planned dental arches; **G**, Positioning of the lower jaw by reference to the ideal virtual occlusion; and H upper and lower jaw surgical virtual planning with native arches

For each patient, an individualized orthodontic treatment was planned (VOP), to simulate orthodontic decompensation and to simulate the pursued occlusion when planning skeletal movements. During the VOP, teeth are positioned in an ideal virtual arch (IVA) (Fig. 9); then, the IVAs were registered to the skeletal base in a manner compatible with the native arches (Fig. 9a–c).

#### Virtual surgical planning (VSP)

In VSP, virtual osteotomies (i.e. LeFort 1, BSSO and/or segmental osteotomy, Fig. 9d, e) are performed on the patient model and subsequently the skeletal segments are repositioned to fulfil both aesthetic and cephalometric criteria (aiming for norm values), within a range of surgical feasibility. First, the upper jaw is repositioned (Fig. 9f) with the native dental arch and upper IVA. Then, the lower jaw is positioned to simulate an ideal virtual occlusion via coordination of both upper and lower IVAs. (Fig. 9g).

After the simulation, a new 3D cephalometry was performed on the virtual setup.

### Treatment

Pre-operatively a passive arch wire was positioned 48 h before surgery. We did not use temporary anchorage devices and did not perform corticotomies.

Surgery was performed without the aid of any additional patient-specific devices (i.e. surgical cutting guides and/or patient-specific implants) besides CAD/CAM surgical splints. Maxillary and mandibular osteosynthesis was obtained using standard titanium miniplates and screws [15, 16]. Patients wore the final surgical splints for 30 days (12–24 h/day) in the post-operative phase. After that, post-operative orthodontic treatment started with replacement of orthodontic wires every 2–3 weeks.

Once the orthodontic treatment was completed, all patients underwent a follow-up CBCT (without braces, at maximum intercuspation) as well as a post-treatment scan of the dental arches. These datasets were used to obtain 3D soft and hard tissue models, on which a new 3D cephalometric study (PostOp) was performed. Mean and standard deviations of the values obtained were considered.

Planned and post-treatment cephalometric measures were compared.

### Statistical analysis

Descriptive statistics (mean and standard deviation) were used to summarize the data, comparing VOSP and PostOp cephalometric analysis. All symmetry measurements were tabulated both as signed and absolute values. The nonparametric Wilcoxon test was used to compare the cephalometric analyses. The significance level was set to α = 0.05.

## Results

VOSP and PostOp 3D Cephalometric data are listed in Table 10.

**Table 10:** VOSP and PostOp 3D cephalometric measurements: Norm values (NORM) [8, 9], Average (Avg), and Standard Deviation (S.D.)

3D Cephalometric measurements	Norm	VOSP Avg	VOSP S.D	PostOp Avg	PostOp S.D	WILCOXONP value
*Sagittal skeletal*						
WITS (mm)	0 ± 2	− 3.24	3.33	− 4.14	2.58	0.033
A/McNamara (mm)	2 ± 2	5.05	2.64	4.1	2.6	0.053
B/ McNamara (mm)	0 ± 2	6.01	3.73	5.68	3.77	0.398
Pog/McNamara (mm)	4	8.05	4.82	7.98	5.94	0.794
Facial Axis Angle (°)	90 ± 3	89.23	4.05	90.35	3.56	0.091
Tweed Mandibular angle (°)	26 ± 4	21.61	4.36	23.6	3.83	0.227
PFH/AFH	0.65–0.75	0.67	0.04	0.64	0.04	0.01
Mean Maxillary length (mm)	n.a	98.97	6.58	98.49	5.79	0.136
△ Maxillary length (mm)	0	1.64	1.26	1.64	1.45	0.984
Mean Mandibular length (mm)	n.a	130.29	11.14	130.71	10.88	0.52
△ Mandibular length (mm)	0	2.52	2.03	1.91	1.71	0.277
Mean Md Body length (mm)	n.a	77.83	8.28	78.62	6.15	0.904
△ Md Body length (mm)	0	2.04	1.95	2.27	1.83	0.845
Mean Gonial angle (°)	n.a	59.03	5.91	57.08	5.86	0.098
△ Gonial angle (°)	0	2.14	1.45	1.93	1.30	0.748
*Frontal skeletal*						
ANS–SagPL (mm)	0	1.2	1.57	1.01	0.92	0.687
A–SagPL (mm)	0	1.41	1.95	0.98	0.72	0.687
B–SagPL (mm)	0	1.94	3.16	1.38	0.94	0.381
Pog–SagPL (mm)	0	2.64	3.96	1.71	1.25	0.952
△ Go-SagPL (mm)	0	3.91	4.66	2.63	2.02	0.334
△ J-SagPL (mm)	0	2.67	3.21	2.22	1.45	0.872
△ Zy-SagPL (mm)	0	1.52	1.39	1.01	1	0.327
*Vertical skeletal*						
Facial ratio	n.a	0.96	0.07	0.97	0.06	0.063
Mandibular ratio	n.a	1.3	0.14	1.3	0.13	0.647
Mean Md Ramus length (mm)	n.a	62.8	7.5	61.21	7.86	0.099
△ Md Ramus length (mm)	0	2.11	1.87	1.81	1.41	0.573
*Sagittal dento-alveolar*						
Incisal protrusion (mm)	4–6	5.68	2.56	6.53	2.63	0.084
UIs/Occlusal plane angle (°)	54 ± 2	59.65	4.94	53.85	4.77	0
IMPA (°)	90 ± 5	91.32	6.93	92.12	6.17	0.243
Overbite (mm)	2.5 ± 2	1.67	0.88	1.89	0.88	0.243
Overjet (mm)	2.5 ± 2	1.93	0.84	3.11	0.8	0.001
Occlusal plane inclination (°)	6 ± 5	5.31	2.77	6.05	3.27	0.777
LIs/B plane angle (°)	25 ± 4	19.28	5.21	19.59	7.04	0.601
*Frontal dento-alveolar*						
UIs-SagPL (mm)	0	1.76	2.39	1.52	1.04	0.355
Lis-SagPL (mm)	0	1.7	2.44	1.77	1.35	0.133
U3-SagPL Mean (mm)	n.a	17.45	1.37	17.58	1.12	0.856
△ U3-SagPL (mm)	0	3.08	3.39	3.04	2.73	0.546
U6-SagPL Mean (mm)	n.a	26.64	1.43	26.74	1.97	0.936
△ U6-SagPL (mm)	0	2.77	2.95	2.54	2.19	0.904
*Vertical Dentoalveolar Analysis*						
△ U3-FH (mm)	0	0.88	1.27	0.93	0.66	0.067
△ U6-FH (mm)	0	1.37	1.85	0.76	0.55	0.199

According to the Sagittal skeletal analysis, in both VOSP and PostOp, jaws resulted on average in a slight biprotrusion, as the distances of points A, B, and Pog from their projections on the McNamara plane were increased when compared to their respective standards. However, the post-operative upper jaw appeared to remain retro-positioned when compared to the planned position; this difference, represented by the A/McNamara distance, is close to statistical significance (*p* = 0.053).

We also observed a slight mean tendency towards skeletal class III in both VOSP and PostOp Wits analysis. When compared, the obtained results appear to be significantly different from the planned ones (*p* = 0.033).

Sagittal dento-alveolar analysis showed that the upper incisor is more protruded in the post-treatment sample, and slightly outside the normal ranges described by James A. McNamara Jr. Accordingly, upper incisor inclination (UIs/Occulsal) is more acute post-treatment (*p* < 0.001).

Regarding the Tweed angle, we obtained an average tendency to hypodivergence, but the norm value still lies within the standard deviation of our cohort.

The strictly frontal values showed a progressively increasing deviation from the sagittal plane in craniocaudal direction, both in VOSP and PostOp (Subnasal Deviation (ANS–SagPL), Maxillary Deviation (A–SagPL), Mandibular Deviation (B–SagPL), Mental Deviation (Pog–SagPL)).

The dento-alveolar frontal analysis showed an increase in the inter-incisal midline deviation in the post-operative outcome compared to planning; however, it was not statistically significant.

The average distance from the sagittal plane and deltas of canine and molars do not show statistically significant differences, and the same applies to the dento-alveolar vertical analysis.

Overbite was slightly less in VOSP, while overjet differed between plan and outcome by around 1 mm; however, only the latter was statistically significant.

## Discussion

3DCA is poorly described in the literature, and no general consensus has been reached regarding standard landmarks and measurements to adopt [8, 11, 13, 17]. To our knowledge, no study describes a 3D cephalometric analysis to be employed as a diagnostic and planning tool with a focus on surgery-first approach.

Although an already established approach, some aspects of SF need further investigation to improve its reliability. In particular, we focused on the description of its shortcomings in VSP reproducibility by means of a 3D cephalometry.

**Fig. 10:**
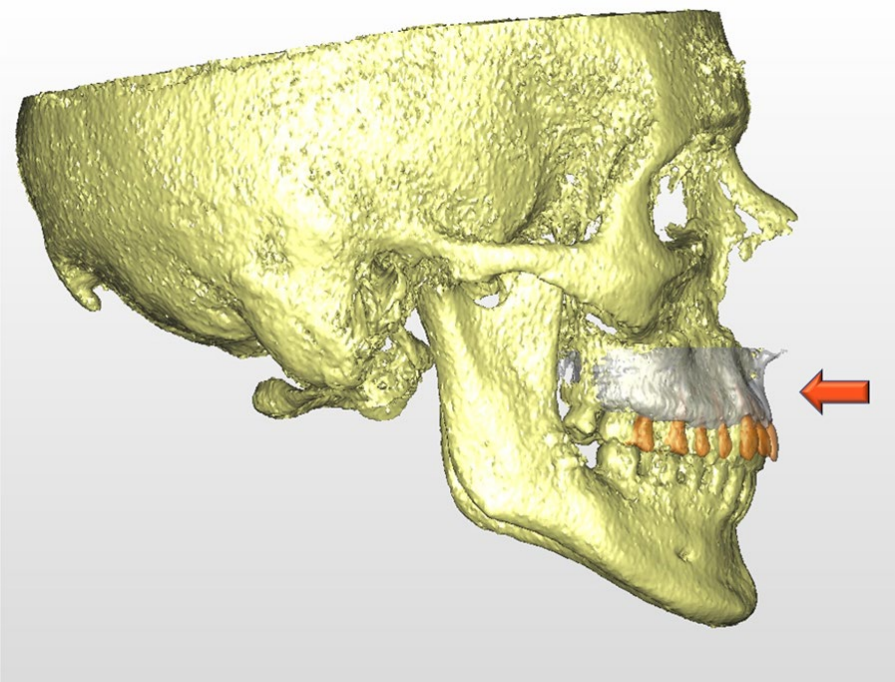
Surgery-first tends to underreach the maxillary advancement: maxilla is retruded by around 1 mm when compared to VSP

In our cohort, the maxillary position compared to VSP was, on average, less advanced by around 1 mm, as demonstrated by the difference in A–SagPL close to statistical significance (*p* = 0.053) (Fig. 10). This result can be partly attributed to the less optimal occlusal stability during the peri-surgical period, partly to a posterior displacement of the A point for intraoperative aesthetic management by means of maxillary reshaping, and partly to posterior condylar sagging in the fossa when guiding the upper maxilla into position [18].

**Fig. 11:**
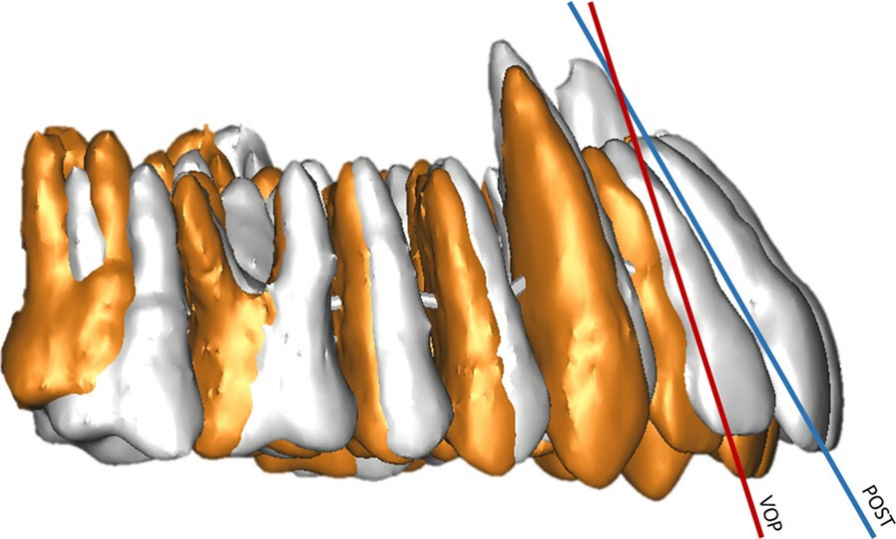
Orthodontists set a positive torque on the central incisors to obtain a correct occlusal relationship

The retruded position of the maxilla is also indicated by the dento-alveolar sagittal analysis score of incisal protrusion, which is greater than planned (5.68 ± 2.56 mm) in the post-operative outcome (6.53 ± 2.63 mm). This can be explained by the inevitable orthodontic compensation that must be performed after maxillary repositioning, a positive torque is set on the central incisors to obtain a correct occlusal relationship. These data find further confirmation in the incisal inclination value, which is more acute post-treatment (53.85 ± 4.77°) compared to the planned one (59.65 ± 4.94°) (Fig. 11), and in the increased post-operative overjet value (VOSP: 1,93 ± 0,84 mm; PostOp: 3,11 ± 0,8 mm). Moreover, the increased occlusal instability is likely to require greater mandibular compensation. Overall, it can be hypothesized that the unpredictability inherent to SF may lead to a slight, clinically imperceptible, under-correction of the dysmorphism.

Although the upper maxilla was less advanced than planned, patients showed, on average, maxillo-mandibular protrusion when McNamara’s plane was considered as reference. This finding is in accordance with the available literature, which reports this trend to be mostly prevalent in younger patients (i.e. juvenile biprotrusion) [19]. Such finding may also be justified by an aesthetic preference for slight biprotrusion in Mediterranean countries, as reported by Pironi et al.[20].

Most patients showed a trend towards skeletal class III in the post-treatment cephalometric analysis, according to Wits index. However, it should be noted that 65% of the sample suffered from skeletal Class III malocclusion before treatment: frequently class III patients tend to show mild class III traits even after surgery, due to surgical and anatomical limitations as similarly reported in the existing literature [21, 22]. All in all, Wits index is a construction measure that cannot represent the only guide within the global case planning, in which the entirety of the aesthetic outcome must be considered; therefore, it is the authors’ opinion that a compromise on the cephalometric outcome can be accepted.

A further interesting finding is that in the cohort analysed, the frontal symmetry tends to decrease in craniocaudal direction. This phenomenon can be again related to the poorer perioperative occlusal stability, which can result in imperfect alignment of the incisor median lines as well as a slight, clinically imperceptible, roll of the maxillary segment. In fact, once the upper maxilla is correctly centred, the other frontal symmetry values may still be more prone to be displaced during the post-operative orthodontic finalization. Another reason for the under-correction of mandibular asymmetries is the possible presence of mild deformities of the mental region which cannot be fully corrected with BSSO surgery alone; thus, the sole cephalometric mandibular midline points are unreliable for an evaluation of the outcome.

A limitation of this study is tied to the non-homogeneity of the sample, due to the fact that the majority of patients were suffering from class III deformity, as it is more likely for skeletal Class III patients to undergo surgery-first approach than Class II or Class I [23]. Relapse patterns in different dysmorphisms can cancel each other on average when considered in a single cohort. However, the statistical analysis used for comparison is free from this shortcoming.

Overall, the significant differences highlighted in this analysis may deserve further analysis in class-specific cohorts for further evaluation.

A further limitation can be found in the lack of an immediately post-operative cephalometric analysis, so to be able to separate an immediate under-realization of VOSP from a relapse caused by muscle tension and dental interferences with bone remodelling during osteotomy healing. This was not performed as, in our workflow, an immediately post-operative CBCT scan is avoided to reduce the radiation exposure of the patient.

## Conclusions

Our 3DCA offers a valid guide for surgeons and orthodontists in planning and analysing the outcome of orthognathic surgery procedures, also in surgery-first approach. However, operator learning curve, global landmarks uniformity and time efficiency in the execution of 3DCA can be object of further evaluation.

This analysis allowed us to evaluate specific aspects which may hinder the predictability of SF approach. Within our sample, the sagittal repositioning of the maxilla represents the main unpredictable factor, as it shows an average tendency to be less advanced compared to the planned position, and therefore leading to an orthodontic compensation via incisal protrusion and proclination. Frontal symmetry is also less controllable, particularly in its mandibular component.

## Data Availability

The datasets used in this study are available by request at the corresponding author.
